# Internal Cumulated Dose of Toxic Metal(loid)s in a Population Residing near Naturally Occurring Radioactive Material Waste Stacks and an Industrial Heavily Polluted Area with High Mortality Rates in Spain

**DOI:** 10.3390/jox15010029

**Published:** 2025-02-08

**Authors:** Manuel Contreras-Llanes, Juan Alguacil, Rocío Capelo, José Luis Gómez-Ariza, Javier García-Pérez, Beatriz Pérez-Gómez, Piedad Martin-Olmedo, Vanessa Santos-Sánchez

**Affiliations:** 1 Research Group in Clinical, Environmental and Epidemiology Social Transformation (EPICAS), Department of Sociology, Social Work and Public Health, University of Huelva, 21007 Huelva, Spain; mcontreras@uhu.es (M.C.-L.); alguacil@uhu.es (J.A.); rocio.capelo@dbasp.uhu.es (R.C.); 2 Research Centre for Natural Resources, Health and Environment (RENSMA), Faculty of Experimental Sciences, University of Huelva, 21007 Huelva, Spain; ariza@dqcm.uhu.es; 3 Consortium for Biomedical Research in Epidemiology & Public Health (CIBER en Epidemiología y Salud Pública—CIBERESP), 28029 Madrid, Spain; jgarcia@isciii.es (J.G.-P.); bperez@isciii.es (B.P.-G.); 4 Department of Chemistry, Faculty of Experimental Sciences, University of Huelva, 21007 Huelva, Spain; 5 Cancer and Environmental Epidemiology Unit, Department of Epidemiology of Chronic Diseases, National Center for Epidemiology, Carlos III Institute of Health, 28029 Madrid, Spain; 6 Andalusian School of Public (EASP), 18011 Granada, Spain; piedad.martin.easp@juntadeandalucia.es; 7 Biosanitary Research Institute of Granada (Ibs. Granada), 18012 Granada, Spain

**Keywords:** Huelva, industrial polluted area, phosphogypsum, metal(loid)s, naturally occurring radioactive material, spatial distribution, toenails, kriging, principal component analysis

## Abstract

Huelva is a city in SW Spain with 150,000 inhabitants, located in the proximity of two heavy chemical industry complexes, the highest naturally occurring radioactive material (NORM) waste (phosphogypsum) stacks of Europe and a highly polluted estuary, with elevated cardiovascular disease and cancer mortality rates. This study analyses the association between cumulated exposure levels to 16 metal(loid)s (Al, As, Cd, Co, Cr, Cu, Fe, Mn, Mo, Ni, Pb, Se, Tl, U, V, and Zn) measured in the toenail of a sample (*n* = 55 participants) of the general control population of Huelva City who were involved in the MCC-Spain study and the spatial proximity patterns to the local polluting sources. Residents of the city of Huelva have higher levels of Fe, Ni, Cr, Se, As, and Co in their toenails compared to the levels found in populations with similar characteristics living in non-polluted areas. Moreover, the highest concentrations of As, Pb, Cd, Mo, and Se were found in toenails of participants living near the NORM waste stack, while the highest Cu, Zn, and Al contents corresponded to people residing near the industrial area. The spatial distribution of most of the metal(loid)s studied appears to be mainly controlled by anthropogenic factors.

## 1. Introduction

The estuary of the Odiel and Tinto rivers (SW Spain), located in the city of Huelva, is documented as one of the major contaminated sites in the world due to the high content of metal(loid)s and radionuclides [1,2].

Industrial and mining environmental pollution have been linked to heart disease, respiratory diseases, and cancer [3]. Huelva City, with 150,000 inhabitants, lies on this Odiel–Tinto Estuary, containing highly toxic elements like As, Cd, Cr, Cu, Ni, Pb, and the most radiotoxic radionuclides (^210^Pb, ^210^Po, and ^226^Ra), associated with the natural ^238^U decay chain [1,2], which may induce adverse health effects [4]. According to previous studies [5,6,7], Huelva City’s heart disease and cancer mortality rates for both women and men are higher than the rest of Spain. Furthermore, the Spanish National Atlas of Mortality reveals a significantly greater standard of mortality ratios in Huelva City during the years 1989 and 2014 among both genders for acute myocardial infarction, heart failure, cerebrovascular diseases, bladder cancer, and other cardiovascular diseases, in addition to lung and breast cancer in men and women, respectively [8]. However, despite the general perception among the general population of a possible link between industrial pollution and the excess of mortality, the issue remains uncertain [5].

This situation has led to social mobilisation and judicial complaints, focusing mainly on the phosphogypsum (PG) stacks, even though there are other sources of exposure. Under pressure from citizens, associations, and environmentalists, judiciary authorities approved the end of the stockpiling of PG in December 2010. In addition, the governmental authorities have approved a restoration protocol plan for the affected saltmarshes by PG stacks, called RESTORE 2030 [9]. Furthermore, this restoration protocol is being re-evaluated due to it not having been approved by the judicial authorities, despite having government approval. Therefore, it is important to carry out a biomonitoring study of metal(loid)s to evaluate the possible health risks of Huelva’s citizens.

Human health status can be monitored by measuring different bioindicators, such as urine, hair, toenails, fingernails, blood, saliva, and milk. Blood, urine, and saliva samples may reflect the short-term exposure (24 h) and are highly influenced by diet [10,11,12]. Nevertheless, hair and nails provide long-term exposure of mineral metabolism, reflecting a period of 6 ± 18 months [13,14,15]. Moreover, hair and nails have several advantages over other bioindicators in the monitoring of metal(loid)s, e.g., being a much less invasive option; they can be easily and inexpensively collected, transported, and stored for a long period before they are analysed, without any changes; the concentration levels are orders of magnitude higher [13,16,17]; and they are the most biosecure option for the analyst who manipulates the sample. Additionally, biomonitoring using toenails reduces the likelihood of external pollution in comparison to fingernails and hair [14,15,18].

Furthermore, geostatistical methods using spatial data are often used for the identification of spatial patterns. Their main objective is to provide unbiased estimates of a sampled variable at non-sampled points [19]. Kriging is a group of geostatistical techniques that interpolate unknown values from nearby known values, thus obtaining a continuous surface of estimated values for the entire area under consideration. This approach is increasingly used for the characterization of the metal(loid)s distribution in soils, as well as for atmospheric or biological sample data [20,21,22]. However, there are few published studies using these spatial analysis techniques to analyse the metal(loid)s signature in toenails. A recent study has been published that analyses the association between the spatial distribution of nail metal(loid)s levels and exposure to pollution sources [22]. Moreover, our research group has previously defined detailed contamination signatures in saltmarsh sediments in the Odiel–Tinto Estuary using geochemical tracers before the restoration protocol plan begins [23].

Therefore, the measurement of exposure at the individual level is a critical aspect in studying the role of these metal(loid)s in the health of the citizens of Huelva. The aim of our research was to analyse the association between the levels of cumulated exposure of 16 metal(loid)s (Al, As, Cd, Co, Cr, Cu, Fe, Mn, Mo, Ni, Pb, Se, Tl, U, V, and Zn) in the toenails of the general population of Huelva and the spatial proximity patterns to the local industrial sources of pollutants.

## 2. Materials and Methods

### 2.1. Study Area

**Figure 1:**
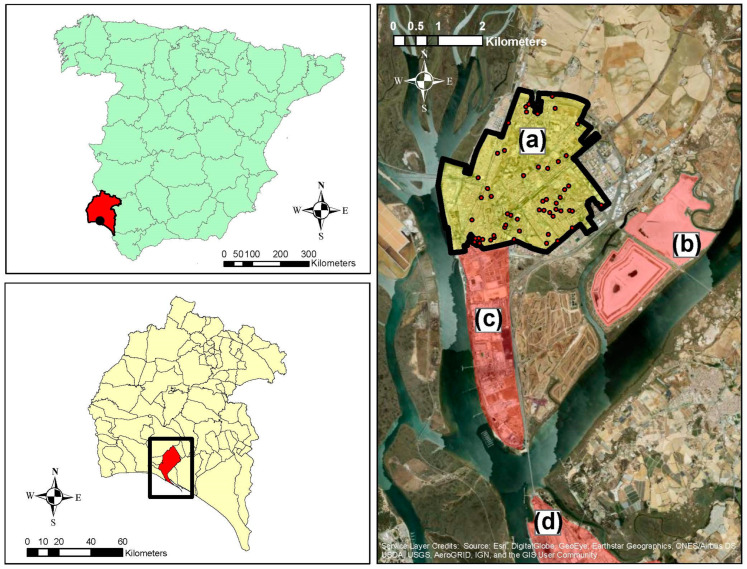
Location map of Huelva City (Spain) [(**a**) residential area of Huelva City (study area); (**b**) PG stacks; (**c**) industrial complex “*Polo Químico de Promoción y Desarrollo de Huelva—Punta del Sebo*”; (**d**) industrial complex “*Nuevo Puerto Palos de la Frontera*”]. Dots represent the locations of participants on a map of the residence area of Huelva City.

We conducted this study in the city of Huelva, located 500 m away from the Odiel–Tinto Estuary. A confluence of anthropogenic stressors gives this estuary a unique character. Both the Tinto and Odiel rivers drain the province of Huelva from north to south through the Iberian Pyrite Belt, one of the most important polymetallic sulphide mining areas in Europe [24,25]. Thus, large amounts of metal(loid)s are transported to the Odiel–Tinto Estuary due to acid mine drainage (AMD) that has been occurring for centuries [26,27]. Moreover, the Odiel–Tinto Estuary receives industrial effluents from factories and wastes located at the edge of the estuary channel, agrochemical drainage due to the intensive agricultural practices from the surrounding farmland, and sewage from Huelva City and other towns located in the estuary (Figure 1) [2,28,29,30]. On the other hand, there is an area over 12,600 m^2^ that is highly polluted with metal(loid)s coming from AMD generated by mineral wastes from an abandoned foundry located in the Odiel marshes, which is exposed to rain and rising river levels during spring tides (Figure 1) [31].

Furthermore, two important chemical parks called “*Polo Químico de Promoción y Desarrollo de Huelva—Punta del Sebo*” and “*Nuevo Puerto Palos de la Frontera*” are also located in the Odiel–Tinto Estuary (Figure 1). The leading Spanish copper and fertiliser producers, a thermal power plant, an oil refinery, etc., are some of the most polluting industries located in these chemical parks [2,29]. Additionally, a vast marsh area of this estuary is occupied by PG (Figure 1), an industrial waste originating from phosphate rock during fertiliser production [32,33,34]. PG was stockpiled on unconsolidated marshes in the Tinto River for a period of 42 years (1968–2010). This area covers 1200 hectares and contains 100 million tonnes, which ranks as one of the most significant in the world [34,35]. These PG stacks contain a wide range of contaminants, for example, organic substances, metal(loid)s and other potentially harmful elements (As, Cd, Cr, Cu, F, Fe, NH^4+^, Ni, P, Pb, S, and Zn), and highly radiotoxic isotopes, such as ^210^Pb, ^210^Pb, and ^226^Ra, from the ^238^U decay series [32,33,34,36,37]. PG is classified as a naturally occurring radioactive material, sometimes known as NORM, which is the term used to describe any radioactive substance that exists naturally in the environment [38].

### 2.2. Study Population

Participants were the population-based controls from Huelva City recruited within the population-based multicase-control (MCC-Spain) study (http://www.mccspain.org), a study designed to explore environmental and genetic factors associated with common tumours or cancers with peculiar epidemiological features, conducted in Spain from September 2008 to December 2013 [39,40]. Participants were aged 20 to 85 years, residing in the catchment area for at least 6 months prior to recruitment. They were randomly selected from the Andalusian Public Healthcare Database (BDU), and were frequency-matched to all cancer and tumour cases, distributed by sex and age (5-year age groups). The ethical and research committees, and each participant, provided written informed consent. The study was performed in accordance with the Declaration of Helsinki.

This study is focused particularly on a representative sample (*n* = 55 participants) of the general population controls of Huelva City involved in the MCC-Spain study.

### 2.3. Toenail Sampling, Laboratory Analyses, and Calibration

After the interview, toenail samples (both feet) from the 55 participants were collected with nail clippers made of stainless steel within 2 weeks of recruitment, placed in a clean plastic bag and stored at room temperature. Moreover, anthropometric data and other information were obtained following the study protocol, which was approved by the recruiting centres.

First, toenail (50–100 mg) samples were washed twice with 2 mL of a 5% (weight/volume) Triton water solution; secondly, they were washed twice using 2 mL of Milli-Q water; thirdly, they were washed twice using 2 mL of acetone; and fourthly, an additional ultrasound treatment (5 min) was conducted. After that, toenails were air-dried and digested with 800 µL of a (4:1) mixture of HNO_3_ and H_2_O_2_ of Ultra Trace Metals grade quality, in a Teflon reactor for microwave-assisted attack. Mineralisation was performed at 400 W, starting from room temperature, ramped up to 160 °C for 15 min, and held for 20 min at this temperature. Finally, the extracts were filtered through a 0.45 µm Polytetrafluoroethylene (PTFE) membrane filter before analysis.

The elemental content of 16 metal(loid)s (Al, As, Cd, Co, Cr, Cu, Fe, Mn, Mo, Ni, Pb, Se, Tl, U, V, and Zn) in toenails was determined by an inductively coupled plasma mass (ICP-MS) system, using an XSeries 2 (Thermo Fisher Scientific Inc., Waltham, MA, USA) spectrometer at the Environmental Bioanalytical Chemistry Unit of Huelva University (Huelva, Spain). Analyses were performed blindly from the case-control status. The measured concentration was adjusted by the equipment, taking into account the dilution factor and sample weight, according to the following formula:

$$Real\;\left(\mu g\cdot {kg}^{-1}\right)=Equipment\;\left(\mu g\cdot {kg}^{-1}\right)*\;\frac{dilution\;factor\;(g)}{sample\;weight\;(g)}$$

The limit of detection for each measured element was obtained from the calibration curve [41].

To control the quality of analysis, the following operations were conducted: (a) 100 mg of human hair was used as reference material (NSC DC73347a) with the purpose of correcting the instrumental variability in each sample batch, with a mean accuracy of 90% maintained along the time ±5%; (b) the ICP-MS response was monitored over time by a measurement of metal(loid)s concentrations at a point on the calibration curve (2 ng mL^−1^) every 20 samples analysed, ensuring an adequate evaluation of the instrument’s response; (c) an instrumental drift correction was performed with the addition of 100 ng mL^−1^ rhodium, as an internal standard, to all of the samples and calibrants, of which those whose response differed ± 10% with respect to the internal standard were measured again; (d) an analysis was conducted every 5 samples of reagents blanks containing 5% (*v*/*v*) HNO_3_ (Suprapur quality), 1% (*v*/*v*) HCl, and Rh 100 ng mL^−1^ in Milli-Q water; (e) an analysis was conducted of duplicate samples every 2.5 h of the sequence; (f) a spiked sample analysis was conducted by spiking the reference materials with the analytes under study (50 ng mL^−1^). Finally, potential interferences from ^98^Mo, ^205^Tl, and ^238^U, regularly existing in nails, were removed by operating the ICP-MS system in helium collision mode (He flow: 4 mL min^−1^); the operative conditions of ICP-ORS-MS are shown in Table 1.

### 2.4. Statistical and Spatial Analyses

A complete descriptive statistical analysis, using mean, geometric mean, median, standard deviation, minimum and maximum concentrations, skewness, and kurtosis, was performed to characterise the metal(loid)s content distribution in toenails. Moreover, in order to determine if the dataset exhibited a normal distribution, the Kolmogorov–Smirnov and Shapiro–Wilk tests were applied to [42].

In addition, Spearman’s correlation coefficient was calculated between the elements studied to assess their potential relationship. Furthermore, a principal component analysis (PCA) was conducted, a statistical method commonly used for the analysis of geochemical and environmental data [43]. This method allows for the reduction of complex data to a discrete number of uncorrelated components. A complex dataset is reduced by creating a small number of uncorrelated principal components, which can describe the entire dataset with minimal loss of original information. A Varimax rotation with Kaiser normalisation was applied, which can maximise the variance of factor loadings across variables for each factor [44]. Furthermore, PCA was developed among the researched metal(loid)s in toenails to evaluate the correlation strength between them and to explore the possible natural or anthropogenic sources of the elements contained in the toenail samples. The statistical significance of a variable was based on *p*-values <0.05 and <0.01. The PCA and Spearman correlations were conducted using IBM SPSS Statistics 26 software.

Spatial variability in the metal(loid)s concentration was studied using kriging, a spatial interpolation method. Kriging was applied to generate continuous surfaces for each of the analysed elements based on data from 55 toenail samples collected from residents in the city of Huelva. This facilitated the visualisation of spatial patterns associated with these elements in unknown areas of the city. Spatial interpolation and distribution pattern maps were created using ArcGIS 10.5 for Desktop software (Esri Inc., Redlands, CA, USA).

## 3. Results

### 3.1. Descriptive Statistics Analysis

The summary of the metal(loid)s contents and descriptive statistics of the toenail samples of the participants collected from Huelva City is listed in Table 2. In all individuals, the Zn arithmetic mean concentration in toenails is much higher (106,042.50 μg·kg^−1^) than other metal(loid)s, such as Al (50,234.39 μg·kg^−1^), Fe (50,132.19 μg·kg^−1^), Cu (4735.88 μg·kg^−1^), Ni (3321.51 μg·kg^−1^), Cr (2196.15 μg·kg^−1^), Pb (827.65 μg·kg^−1^), and As (241.48 μg·kg^−1^). A similar result was achieved between the arithmetic mean and the geometric mean values, denoting a similar tendency and spatial variations of the data in Huelva City. Zn had the highest geometric mean concentration (102,480.18 μg·kg^−1^), followed by Al (44,229.43 μg·kg^−1^) and Fe (38,544.96 μg·kg^−1^). The geometric mean concentrations of elements in toenails decreased in the following order: Zn, Al, Fe, Cu, Ni, Cr, Se, Pb, Mn, As, V, Mo, Co, Cd, U, and Tl. The arithmetic mean concentrations decreased in the same order, only appearing to have a small discrepancy with Mn.

### 3.2. Spearman Correlation Coefficient Analysis and Principal Component Analysis (PCA)

Spearman’s correlation coefficients of elements in toenail samples from Huelva City are shown in Table 3. Most of the metal(loid)s pairs displayed positive relations, which were significant, at 95%, and/or a higher confidence level (99%) in some cases, with correlation coefficients above 0.7.

PCA was conducted in order to identify the likely contributing factors towards the metal(loid)s concentrations, and thereby define which metal(loid)s may have a common origin. For the PCA (Table 4), participants with metal(loid)s contents in the toenail samples above the LOD were included (*n* = 55). Six components/factors (PC1, PC2, PC3, PC4, PC5, and PC6) with eigenvalues >1 (Kaiser criterion), were obtained according to results of the initial eigenvalues [44], which were extracted from the study area, explaining practically 70% of total variance. The first principal component (PC1) is formed for Mn, V, Al, Fe, Co, and U, explaining 27% of the total variance. Zn, Cr, and Pb were the components of the second principal component (PC2) and explained 11.2% of the total variance. The third principal component (PC3) is characterised by Cd, As, and Cu, which described only 9.6% of the total variance. The fourth principal component (PC4) is formed for Mo and Tl, explaining 7.8% of the total variance. Moreover, Se was the element with the highest positive PCA loading of the fifth principal component (PC5), although Pb has a high negative loading (−0.517); they were responsible for 7.2% of total variance. Finally, the sixth principal component (PC6) represented 6.4% of the total variance, which is only dominated by Ni.

### 3.3. Spatial Variability

Figure 1 shows the locations of the two industrial complexes, PG stacks and the geocoded addresses of participants. According to previous studies, Huelva is situated downwind from these main emission sources, where air pollution and the diffusion of metal(loid)s are of major concern [45,46,47,48,49,50,51].

**Figure 2:**
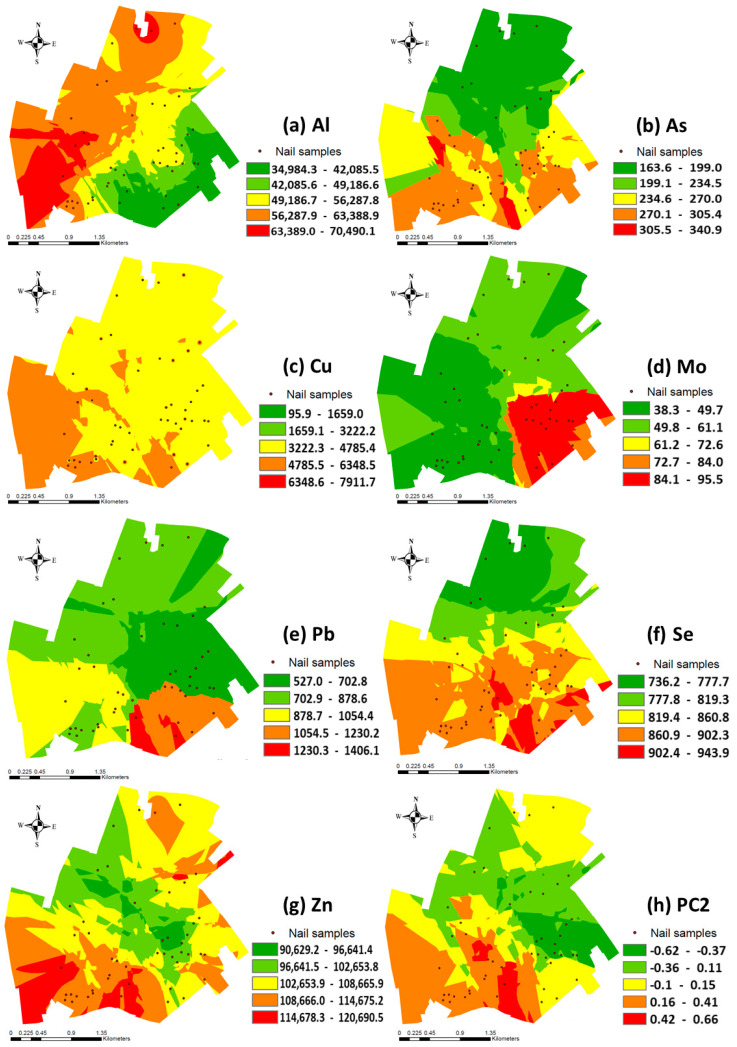
Spatially interpolated surfaces of metal(loid)s [(**a**) Al; (**b**) As; (**c**) Cu; (**d**) Mo; (**e**) Pb; (**f**) Se; (**g**) Zn; (**h**) PC2]. Dots represent the locations of participants on a map of the city of Huelva.

Figure 2 illustrates the spatially interpolated surfaces obtained by the kriging interpolation method of Al, As, Cu, Mo, Pb, Se, and Zn, and the combined PC2 from PCA, which serves as an example to observe how the distribution of combined PCA denotes similar signatures as those observed in the interpolated surfaces of each element studied. Al and Cu (Figure 2a,c) show higher values of these elements in the area of the city closest to the industrial pole. In Figure 2c, for Cu, only areas near industrial activities revealed the major concentrations, while the area far downwind from there showed relatively minor Cu values. Similarly, Al presents the highest concentration near the industrial activity area, although a small hotspot with a high concentration of Al was identified in the north of the city. Nevertheless, the concentration in toenails was generally low compared to other places, and especially close to the PG stacks (Figure 2a). Furthermore, As, Se, and Zn show a spatial pattern in which the values obtained both in the vicinity of the industry and in the vicinity of the PG stacks stand out (Figure 2b,f,g). The spatial distribution of As revealed that the highest concentration is dispersed near to the industrial complexes and PG stacks (Figure 2b). Meanwhile, a small hotspot with high As levels was located far away in the northwest (but not the immediate neighbourhoods), although the concentrations remained generally low in the studied area. Selenium also presents the highest measurements in the same area of Huelva; nevertheless, the concentrations of Se in areas far away had relatively low concentrations (Figure 2f). In the same way, Zn shows the highest values in the area of the city closest to the industrial pole and PG stacks, whereas some small patches had relatively high Zn concentrations in the northeast (Figure 2g). On the other hand, the spatial distribution of Mo and Pb (Figure 2d,e) shows higher values in the area close to the PG stacks. In Figure 2e, for Pb, neighbourhoods located nearby to the industrial activity area denoted relatively elevated concentrations, but participants with the major lead level in toenails were located downwind, close to the PG stacks. Other places away from the PG stacks had relatively minor nail levels of the measurements. Similarly, participants residing close to the PG stacks had the highest concentrations of Mo (Figure 2d). Meanwhile, Mo concentrations were lower in areas close to the industrial poles. Finally, PC2 denotes a similar pattern distribution compared to them in the interpolated surfaces of the individual elements that form it, being Cr, Pb, and Zn (Figure 2h). Overall, participants located nearby the industrial complexes and PG stacks shower relatively major measurements of the elements studied than that rest of Huelva City.

## 4. Discussion

Our study revealed that the mean levels of Ni, Cr, Se, As, and Co were considerably higher than those of unpolluted areas with similar population characteristics (culture, diet, and lifestyle). The results of our study show correlations between the elements analysed in the toenail samples of the residents of the city of Huelva. Many of these elements are distributed in a similar way, finding a spatial pattern in their distribution, with higher values of metal(loid)s in the vicinity of the PG stacks and/or industrial complexes. The results are novel and provide insights into the exposures of a population located close to an extremely polluted estuary.

According to other studies among populations residing near polluted areas (Table 5), our population revealed that Zn has the maximum mean levels in toenails, followed by Al, Fe, Cu, Ni, Cr, Mn, Se, Pb, As, V, Mo, Co, Cd, U, and Tl [52,53,54,55,56,57,58,59,60], although this order differs from other similar studies [61,62,63]. Moreover, these levels were found to be similar to the other studies carried out in particularly contaminated areas [53,54,55,56,57,59,63]. Furthermore, it was observed that our results are similar to other populations living in polluted areas with similar conditions, as in the case of Kima (Egypt), which includes a fertiliser industry, industrial polluted areas, and heavy traffic [57]. Moreover, similar results to a population residing close to a mine waste dump in the Zambian Copperbelt, an area environmentally affected by copper mining, were also found [55]. However, the discrepancies between our results and some of the compiled research [61,62], shown in Table 5, might be due to the differences in environmental area, sanitation and statistical characteristics of human populations, predominant sources, and variance in the degree of exposure of people to these metal(loid)s. On the other hand, in other studies carried out in unpolluted areas with similar population characteristics (culture, diet, and lifestyle), such as the island of Mallorca (Spain) [59] and Forlì (Italy) [61], the mean levels of Fe, Ni, Cr, Se, As, and Co were quite minor compared to those of our data. These results suggest that the people living in Huelva bioaccumulate these metal(loid)s.

These results are supported by previous studies, such as a study focused on the urinary levels of heavy metals, such as As, Cd, Cr, Cu, and Ni, in children from Huelva, which revealed that the mean concentration of Cd was higher than rates reported in other studies conducted in Europe [64]. Similarly, another study denoted the cognitive behaviour of children living in the city of Huelva with high levels of Cd in both their hair and urine [65]. Another recent explorative study associated the environmental exposure to heavy metals and neurobehavioural performance in the children of Huelva [66]. Furthermore, industry workers show high levels of arsenic and other metals, even after taking into account fish and seafood consumption [67]. Less than 500 m from the city are the highest NORM waste stacks of Europe, and an industrial park including a copper smelter, a petrol refinery, and a fertiliser production plant, among others (Figure 1) [2,29,32,34]. In addition, the local river carries acid mine drainage from the mining activity upstream [1,2]. There are various reports showing the impact of the industrial pollution on the local river and the nearby estuary and marshes [29,30].

Spearman correlation analysis among levels of the different elements may reflect the combined effect of common sources of exposure (e.g., environmental, occupational, and dietary) and common toxicokinetic activities during absorption, transport, and bioaccumulation in toenails. Investigating how these sources contribute to exposure and their influence on metal(loid)s mixtures is important for assessing health outcomes. Additionally, PCA was used as a statistical process for reducing the dimensionality of correlated heavy metal(loid)s [43].

Mn, Al, U, Co, V, and Fe were significantly correlated with each other at a 99% or higher confidence level (Table 3). A high correlation was obtained between the following metal(loid)s pairs: V-Mn (*r* = 0.713), V-Al (*r* = 0.696), and V-Fe (*r* = 0.673). Moreover, Mn-Fe (*r* = 0.777) and Mn-Al (*r* = 0.591) showed a strong and moderate correlation, respectively. Finally, Co-V (*r* = 0.623) also revealed a strong correlation. These strong correlations indicate that a general trend of high concentration in one element should indicate high concentrations in the others. Additionally, these loadings denoted that these elements come from the same source, or that they belong to the same source but with diverse origins, while a moderate correlation was observed among the U-V (*r* = 0.527), U-Co (*r* = 0.526), and the other metal(loid)s pairs (U-Mn, U-Al), as well as weakly correlated U-Fe (*r* = 0.386), revealing a different source (Table 3). These results may indicate a common source, probably with various origins, which were mainly a result of mixed natural and anthropogenic activities, mutual dependence, or identical behaviour during transportation [68]. On the other hand, Mn and V were very strongly associated, and Al, U, Co, and Fe were strongly associated; these components characterised factor one (PC1), which explained 27% of the total variance (Table 4). This means that all cases have the same (positive) sign and strong association, indicating that all of the elements interact in the same manner in the structure of the nails (Table 4). Furthermore, all of the results corresponding to PC1 have the same (positive) sign, indicating that each element influences PC1 in the same manner. Some previous studies based on the air quality of Huelva confirmed that Al, Mn, and Fe emissions are related to crustal sources [45,46,47,48,49,50,51]. Additionally, Al, Mn, and Fe levels were also higher in industrial areas, and the spatial distribution may have been controlled by both anthropogenic and natural causes [62].

The interpolation analysis revealed the information shown in Figure 2a, which demonstrates the highest Al concentration, near and far from the industrial hubs, which could be explained by different origins, the industrial activities, and the crustal source. Moreover, these previous studies based on the air quality of Huelva also attributed most of the emissions to V and Co in Huelva City, with the crude oil refinery located in the “*Nuevo Puerto Palos de la Frontera*” industrial estate [45,46,47,48,49,50,51]. On the other hand, the PG stack and the phosphoric fertilisers’ activities could be considered the main sources of U [32,33,34,36,37].

The second factor (PC2) was strong for Zn and moderated for Cr and Pb; the loading indicates that each element influences PC2 in the same manner due to the same positive sign (Table 4). This showed that the availability of Cr in the toenail samples would slightly influence both Pb and Zn and their respective environmental and human-induced effects. Furthermore, positive weak correlations were also observed between Cr-Pb (*r* = 0.393) and Cr-Zn (*r* = 0.286) at *p* < 0.01 and *p* < 0.05, respectively (Table 3). PC2 denoted a mixture of sources because these elements were not correlated with both themselves and other elements, indicating that they may have had different origins. These components are suspected to emanate from the disposal of industrial waste, industrial air-borne emissions, and high traffic near the area, along with roadside dust. According to previous studies on the air quality of Huelva, Pb and Zn emissions are associated with a Cu smelter plant located in the left bank of the Odiel river in the “*Polo Químico de Promoción y Desarrollo de Huelva—Punta del Sebo*” industrial complex; meanwhile, Cr is traced to traffic emissions [45,46,47,48,49,50,51]. The results of Spearman’s linear correlation quantified our visual interpretations of the associations between the vicinity to industrial complexes and the levels of these elements (Table 3).

The spatial distribution of Pb (Figure 2e) shows higher values in the area close to the PG stacks, although neighbourhoods residing close to the industrial complexes revealed relatively high concentrations of measures. This finding could be associated with the impact of emissions from industrial contaminating sources, in addition to the emissions generated near PG stacks, which are particularly linked to traffic emissions, since the roads with the highest volume of vehicles are there, including heavy traffic. On the other hand, Zn shows the highest values in the area of the city closest to the industrial pole, whereas some small patches had relatively high Zn concentrations in PG stacks (Figure 2g). This fact confirmed that Zn emissions were released from the industrial pole.

On the other hand, Cd and As were strongly positive and Cu was moderately associated; these components characterised factor three (PC3), which explained only 9.6% of the total variance (Table 4). The association among these elements indicates the environmental influence of these elements on nail samples and possibly different sources of pollution. Moreover, there is no significant correlation between metal(loid)s pairs in PC3, and there is also no correlation with the other elements (Table 3). This result revealed that those elements are generated in different sources at the same time and show different behaviours during transportation into the toenails [68]. A possible source for these components is industrial emissions [69]. Moreover, there is a strong metallurgical activity located in the “*Polo Químico de Promoción y Desarrollo de Huelva—Punta del Sebo*” industrial estate, which releases As and other metal(loid)s [45,46,47,48,49,50,51]. Additionally, Cu^2+^ and As could be the result of industrial emissions from both the fertiliser factory and PG waste, as due to the proximity to the industrial pole and the northwest wind direction, Huelva is exposed to heavy atmospheric deposition of these elements [32,33,34,45,46,47,48,49,50,51]. On the other hand, most of the Cd emission in the city of Huelva was attributed to the petrochemical industries, as well as the TiO_2_ pigment industries, which also release a small amount of Cd and As; both are located in the “*Nuevo Puerto Palos de la Frontera*” industrial complex [45,46,47,48,49,50,51].

The spatial distribution of As is in accordance with previous statistical results, since As is generated from different sources, such as the Cu smelter, fertiliser industries, and the oil refinery, all of them located southeast of the city of Huelva (Figure 2b). Conversely, kriging maps show upper values of Cu in the area of the city closest to the industrial complexes, downwind of the copper smelter activities, revealing this as a unique source.

The fourth principal component (PC4) explained 7.8% of the total variance and included Mo and Tl, which were very strongly and moderately associated, respectively (Table 4). This positive association identifies a moderate relationship between these elements. In addition, toenail thallium and molybdenum concentrations were weakly correlated, with a moderate Spearman coefficient. Moreover, these elements were not correlated with other elements (Table 3), revealing different sources in the Huelva area. The PG stacks and the emissions from industrial activities, such as phosphoric fertilisers, might be assumed as the sources of Mo and Tl [32,34]. The spatial distribution of Mo (Figure 2d) shows higher values, mainly in the area close to the PG stacks, which is in accordance with the previous statement.

In addition, the fifth principal component (PC5) was dominated by Se and Pb, with a strongly positive and moderately negative association, respectively (Table 4). This fact indicates that these elements interact in the opposite manner in the structure of the nails, revealing an antagonistic effect between Se and Pb. Some studies have demonstrated that selenium has a great toxicant affinity and forms biologically inert complexes [70,71], thus potentially protecting against metal(loid)s bioaccumulation. Moreover, selenium comes from a different source than the other studied elements, since it was not correlated with any of the other elements (Table 3). The principal route of human exposure to selenium is through the diet, food, and drinking water, although smoking is an inadvertent inhalation exposure route to selenium [72].

Furthermore, Se shows a spatial pattern in which the values are obtained both near the industry and in the vicinity of the PG stacks; it reveals some influence by the industrial emission (Figure 2f). No studies have been found in the bibliography that verify the contribution of Se by industrial activity, and because this is an essential element that usually has a protective vision for health, it is not usually taken into account in studies of biomonitoring, although its presence in excess can produce toxicity.

Finally, the sixth principal component (PC6) explained 6.4% of total variance and is characterised by high-loading of nickel (Table 4). In addition, only a moderate positive correlation, at *p* < 0.01, was found between Ni-Co (*r* = 0.536), suggesting that both components may have the same origin (Table 3). The petrochemical industries located in the “*Nuevo Puerto Palos de la Frontera*” industrial area are the main emission sources of Ni [45,46,47,48,49,50,51].

### Strengths and Limitations

Our findings must be interpreted in light of some limitations. First, this study is based on a small population of 55 participants among 150,000 inhabits, and this fact may limit our statistical power. Second, the participants may have been exposed to metal(loid)s from other confounding variables, such as sociodemographic factors, lifelong retrospective environmental exposures, occupational history, traffic, or diet, which should be addressed in upcoming investigations. Nevertheless, the loading of the main elements of the principal sources would not have been impacted, since we considered multiple metal(loid)s in the PCA. Third, PCA and Spearman’s correlation analysis are techniques used to identify the probable source; however, the interpretation of the results depends on the knowledge and experience of the expert. The identification of the different patterns requires a careful interpretation based on possible local sources of contamination (both anthropogenic and natural sources), different exposure routes, and other specific local characteristics. Thus, we relied on various previous studies that have been conducted to characterise the quality of Huelva City’s air [45,46,47,48,49,50,51], the industrial impact on the local river and the nearby estuary and marshes [29,30], the acid mine drainage from the mining activity upstream [1,2] and other studies on metal(loid)s sources’ identification [2,29,32].

Regardless of the exposed limitations, our research also has several strengths. First, this is the first study developed on Huelva’s citizens living in an extremely polluted area which analyses the link between potential industrial pollution exposure and metal(loid)s concentrations in toenails using statistical techniques of spatial analysis, such as kriging. Second, this study used ICP-Ms analysis, which has low LODs for most metal(loid)s studied, and may have identified low-level exposures to uranium, lead, thallium, and arsenic. Thus, we obtained data from a representative sample of Huelva’s population, where we may potentially have detected 16 metal(loid)s in toenails. Normally, similar previous studies determine a limited number (one to six elements) of potentially harmful elements because they used characterisation techniques with high LODs [22]; however, we have selected and analysed a total of sixteen heavy metal(loid)s. Third, toenails were used as a bioindicator, a non-destructive method widely accepted as an internal exposure marker, which could reflect the long-term patterns of mineral metabolism, reflecting a period of 6 ± 18 months [13,14,15]. Fourth, as differential effects may be potentially induced by environmental agents’ exposures to different lengths of time [73], the participants’ inclusion criterion was residing in Huelva City for at least 6 months. Thus, we warrant to estimate the long-term environmental exposition time for the study population. Fifth, demographic variables, such as age, gender, and socioeconomic status, have been included to influence potential exposure and, consequently, the elements’ levels in toenails [13,14,15]. These variables were controlled for in the analysis. Sixth, PCA is an unbiased approach to assess the inter-relationship of the elements identified in toenail samples [14,15,43,56,60]. Seventh, applying Kriging geostatistical techniques enhanced our understanding of the association between vicinity to contamination sources and elements’ levels in toenails of Huelva’s citizens [20,21,22]. Eighth, the specific internal dose of cumulated toxic metal(loid)s concentration signatures obtained in this research could be used as a reference to identify the influence of future interventions on current contamination sources in the Odiel–Tinto Estuary. These findings are critical for measuring the effect of the RESTORE 2030 plan [9], which is currently being re-evaluated by judicial authorities [10]. To the best of our knowledge, there are no published references nor permissive levels of metals in toenails to compare whether the absolute concentrations found in our study are within permissive levels.

## 5. Conclusions

Residents of the city of Huelva have higher levels of Fe, Ni, Cr, Se, As, and Co in toenails compared to the levels found in populations with similar characteristics living in non-polluted areas. Our results indicate that citizens of Huelva residing in close-proximity to phosphogypsum stacks, a NORM waste stack, and an industrial heavily polluted area denote major contents of many metal(loid)s in toenails. The concentrations of As, Pb, Cd, Mo, and Se were high in the population living near the PG stacks, whereas Cu, Zn, and Al levels were high in people residing near the industrial area. These findings are a crucial first step towards understanding the signature of cumulated human toxic metal(loid)s’ concentration in this population, and they raise significant interrogations concerning the effect of the industry on health in this city. The results bring to light the real impact of the industry on the health of the citizens of Huelva City, particularly, the residents residing closest to the pollution sources, a fact that had not yet been demonstrated. The cumulated exposure at the individual level is a critical aspect in the study of the role of these metal(loid)s on the health of the inhabitants of Huelva. Furthermore, this study might serve as a reference to assess the impact of the restoration plan (RESTORE 2030) for the affected area in the Odiel–Tinto Estuary. These specific internal doses of cumulative toxic metal(loid)s concentration patterns could also be used as a reference to evaluate the influence of future interventions in other similarly contaminated areas.

## Figures and Tables

**Table 1:** Operative conditions of ICP-ORS-MS.

Forward power	1500 W
Plasma gas flow	15 L min^−1^
Auxiliary gas flow 1 L min^−1^	1 L min^−1^
Carrier gas flow	0.15 L min^−1^
Sampling depth 7 mm	7 mm
Sampling and skimmer cones	Ni
H_2_ flow 4	4 mL min^−1^
Nebuliser	Microflow (ESI)
Torch	Shield (with long-life platinum shield plate)
Qoct −18 V	18 V
Qp	−16 V
Points per peak	1
Integration time	0.3 s per isotope
Replicates	1
Isotopes monitored for total metals in nail	^27^Al, ^75^As, ^114^Cd, ^59^Co, ^52^Cr, ^63^Cu, ^56^Fe, ^55^Mn, ^98^Mo, ^58^Ni, ^208^Pb, ^80^Se, ^205^Tl, ^238^U, ^51^V, ^64^Zn
Dead time of detector	47 s

**Table 2:** Concentrations of detected metal(loid)s in toenails (μg·kg^−1^).

	*n* ^a^	AM ± SD ^b^	Median	GM (GSD) ^c^	Min	Max	Skewness	Kurtosis
Al	55	50,234.39 ± 26,471.07	42,916.47	44,229.43 (1.66)	12,601.90	130,583.32	1.13 ± 0.32	0.86 ± 0.63
As	54	241.48 ± 177.86	190.91	207.50 (1.64)	99.24	1085.34	3.09 ± 0.32	11.04 ± 0.63
Cd	54	19.53 ± 16.61	16.23	16.11 (1.79)	3.80	111.46	3.94 ± 0.32	19.01 ± 0.63
Co	55	35.65 ± 29.06	28.87	28.84 (1.87)	8.47	173.57	2.97 ± 0.32	10.83 ± 0.63
Cr	55	2196.15 ± 2442.15	1521.12	1388.91 (2.71)	103.50	14,195.03	2.90 ± 0.32	10.89 ± 0.63
Cu	54	4735.88 ± 1065,34	4663,39	4624.11 (1.25)	2667,66	7972.01	0.87 ± 0.32	1.63 ± 0.63
Fe	55	50,132.19 ± 39,323.87	35,329.31	38,544.96 (2.06)	11,039.44	164,373.54	1.49 ± 0.32	1.61 ± 0.63
Mn	55	870.41 ± 1292.21	590.09	608.10 (2.12)	145.97	9608.01	5.98 ± 0.32	40.20 ± 0.63
Mo	55	56.76 ± 53.69	40.67	47.61 (1.68)	12.59	410.53	5.52 ± 0.32	35.95 ± 0.63
Ni	55	3321.51 ± 6407.36	2127.67	2054.46 (2.37)	257.05	48,325.41	6.65 ± 0.32	47.21 ± 0.63
Pb	55	827.65 ± 894.39	579.75	657.42 (1.81)	175.79	6486.15	5.09 ± 0.32	30.64 ± 0.63
Se	55	841.40 ± 180.95	815.97	822.97 (1.24)	395.14	1463.82	0.86 ± 0.32	2.10 ± 0.63
Tl	55	4.49 ± 1.91	3.98	4.15 (1.49)	1.60	11.57	1.44 ± 0.32	3.21 ± 0.63
U	52	6.46 ± 5.41	4.76	5.08 (1.94)	1.24	25.16	2.17 ± 0.32	4.38 ± 0.63
V	55	78.41 ± 64.85	59.49	64.30 (1.80)	17.84	409.82	3.25 ± 0.32	13.20 ± 0.63
Zn	55	106,042.50 ± 28,459.60	100,163.44	102,480.18 (1.30)	60,198.53	177,989.35	0.66 ± 0.32	−0.19 ± 0.63

^a^ Number of samples whose concentration was above the LOD. Values reported in the table are for those participants who were above the LOD. ^b^ AM ± SD, arithmetic mean ± standard deviation. ^c^ GM (GSD), geometric mean (geometric standard deviation).

**Table 3:** Spearman’s correlation coefficients of detected metal(loid)s in the toenail samples.

	Mn	V	Al	Fe	Co	U	Zn	Cr	Pb	Cd	As	Cu	Mo	Tl	Se	Ni
Mn	1															
V	0.713 **	1														
Al	0.591 **	0.696 **	1													
Fe	0.777 **	0.673 **	0.474 **	1												
Co	0.564 **	0.623 **	0.475 **	0.514 **	1											
U	0.471 **	0.527 **	0.467 **	0.386 **	0.526 **	1										
Zn	0.003	−0.059	0.025	−0.136	−0.044	0.086	1									
Cr	0.394 **	0.453 **	0.409 **	0.405 **	0.217	0.330 *	0.286 *	1								
Pb	0.461 **	0.522 **	0.466 **	0.336 *	0.468 **	0.443 **	0.101	0.393 **	1							
Cd	0.318 *	0.321 *	0.155	0.287 *	0.202	0.389 **	−0.037	0.240	0.238	1						
As	−0.035	−0.020	−0.177	0.023	0.077	−0.008	−0.018	−0.117	−0.024	0.216	1					
Cu	0.459 **	0.373 **	0.330 *	0.296 *	0.491 **	0.376 **	0.168	0.256	0.440 **	0.277 *	0.096	1				
Mo	0.334 *	0.468 **	0.253	0.408 **	0.462 **	0.233	0.144	0.063	0.185	0.075	0.029	0.274 *	1			
Tl	0.343 *	0.274 *	0.175	0.216	0.361 **	0.281 *	0.064	0.089	0.288 *	0.066	0.181	0.268 *	0.225	1		
Se	0.002	−0.147	−0.148	−0.042	0.071	0.151	0.169	−0.082	−0.104	−0.102	0.245	0.241	0.095	0.135	1	
Ni	0.491 **	0.334 *	0.451 **	0.301 *	0.536 **	0.285 *	−0.200	0.139	0.275 *	−0.017	−0.264	0.254	0.141	0.217	−0.074	1

* Correlation is significant at the 0.05 level (2-tailed). ** Correlation is significant at the 0.01 level (2-tailed).

**Table 4:** Principal component analysis (PCA) results of metal(loid)s in the toenail samples.

	PC1	PC2	PC3	PC4	PC5	PC6
Mn	**0.880**	0.005	0.093	−0.043	0.050	−0.015
V	**0.845**	−0.055	0.115	0.368	−0.014	−0.088
Al	**0.773**	0.151	−0.124	−0.019	−0.120	0.049
Fe	**0.762**	0.103	0.034	0.244	−0.105	−0.072
Co	**0.724**	−0.093	0.112	0.081	−0.040	0.273
U	**0.703**	0.176	0.005	−0.013	0.234	−0.056
Zn	−0.088	**0.800**	0.046	−0.047	−0.001	−0.127
Cr	0.224	**0.652**	−0.062	−0.007	0.122	−0.124
Pb	0.108	**0.530**	0.152	0.117	**−0.517**	0.271
Cd	−0.041	0.078	**0.821**	−0.066	−0.098	−0.064
As	0.112	−0.093	**0.794**	0.029	0.190	−0.092
Cu	0.320	0.323	**0.453**	0.268	0.270	0.326
Mo	0.080	−0.152	0.069	**0.902**	−0.088	−0.116
Tl	0.208	0.204	−0.137	**0.545**	0.257	0.164
Se	−0.030	0.110	0.156	0.077	**0.888**	0.037
Ni	−0.022	−0.200	−0.141	−0.039	−0.023	**0.841**
Eigenvalues	4.318	1.793	1.537	1.246	1.145	1.028
Total of variance (%)	26.99	11.20	9.61	7.79	7.16	6.43
Cumulative variance (%)	26.99	38.19	47.80	55.58	62.74	69.17

The values indicated in bold are crucial for explaining the PCA, as a larger number signifies a more significant contribution.

**Table 5:** Comparison of metal(loid)s levels (μg·g^−1^) in general environmentally exposed populations found in previous studies worldwide in human toenails.

Researchers	Biological Sample; *n* ^a^	Study Area and Sources	Al	As	Cd	Co	Cr	Cu	Fe	Mn	Mo	Ni	Pb	Se	Tl	U	V	Zn
Our study	Toenails; *n* = 55	People living close to an extremely polluted estuary in Huelva, Spain	50.2	0.24	0.02	0.04	2.195	4.74	50.1	0.87	0.06	3.32	0.83	0.84	0.005	0.007	0.08	106.0
Bechtold et al., 2020 [52]	Toenails; *n* = 489	Population living near a municipal solid waste incinerator in Modena (Italy)	N.M.	N.M.	0.02 ± 0.02	N.M.	1.33 ± 2.92	4.10 ± 3.4	N.M.	N.M.	0.39 ± 0.57	1.04 ± 3.08	0.86 ± 1.48	0.49 ± 0.09	N.M.	N.M.	N.M.	95.6 ± 34.6
Butler et al., 2019 [53]	Toenails; *n* = 521	A region with ferroalloy industry in Brescia, Italy	70	N.M.	N.M.	N.M.	0.15	2.66	N.M.	0.19	N.M.	N.M.	0.10	N.M.	N.M.	N.M.	N.M.	110
Coelho et al., 2014 [54]	Toenails; *n* = 122	Villages near the Panasqueira mine, in central Portugal	N.M.	0.65 ± 0.56	0.05 ± 0.04	N.M.	2.17 ± 2.41	N.M.	N.M.	2.84 ± 3.17	N.M.	4.12 ± 9.20	1.25 ± 1.33	0.63 ± 0.35	N.M.	N.M.	N.M.	136.49 ± 80.72
Di Ciaula et al., 2020 [61]	Toenails; *n* = 62	Population living in an urban area and close to waste incinerators in Forlì, Italy	166.48 ± 50.42	0.01 ± 0.01	0.03 ± 0.004	0.04 ± 0.04	4.82 ± 3.88	6.34 ± 0.70	360.08 ± 126.57	4.40 ± 1.23	0.00	2.23 ± 1.51	0.32 ± 0.13	0.01 ± 0.005	0.00	0.00	0.19 ± 0.11	96.27 ± 9.42
Nakaona et al., 2020 [55]	Toenails; *n* = 40	Community living near a mine waste dump in the Zambian Copperbelt	N.M.	N.M.	0.1 ± 0.002	1.0 ± 0.02	0.6 ± 0.08	29.6 ± 4.8	N.M.	12.0 ± 2.02	N.M.	1.7 ± 0.14	4.8 ± 0.53	N.M.	N.M.	N.M.	N.M.	172 ± 27.4
Ojekunle et al., 2022 [62]	Toenails; *n* = 38	Neighbourhood close to a dumpsite waste in Nigeria	N.M.	N.M.	2.1 ± 4.5	2.9 ± 6.9	55.6 ± 35.2	95.4 ± 45.4	N.M.	108.5 ± 167.8	N.M.	156.0 ± 172.9	36.6 ± 89.4	N.M.	N.M.	N.M.	N.M.	354.3 ± 333.8
Przybylowicz et al., 2012 [56]	Toenails; *n* = 42	Adults from environmentally exposed areas at living in the same area (Krakow)	N.M.	N.M.	0.37 ± 0.41	0.04 ± 0.06	2.44 ± 1.88	4.80 ± 1.25	42.9 ± 22.4	N.M.	N.M.	2.73 ± 1.24	0.66 ± 0.15	N.M.	N.M.	N.M.	N.M.	121.4 ± 30.4
Rashed and Hossam, 2007 [57]	Toenails; *n* = 115	Adults from environmentally exposed areas at Aswan, Egypt	N.M.	N.M.	1.0 ± 0.4	N.M.	N.M.	16.2 ± 1.5	N.M.	N.M.	N.M.	N.M.	22.3 ± 3.6	N.M.	N.M.	N.M.	N.M.	158 ± 22
Slotnick et al. 2004 [58]	Toenails; *n* = 163	Residing in a highly industrialised Area in Detroit, USA	26.9 ± 21.6	0.10 ± 0.22	0.64 ± 0.79	0.17 ± 0.86	1.91 ± 1.66	5.05 ± 4.64	N.M.	N.M.	0.60 ± 1.15	32.89 ± 68.97	0.74 ± 1.23	0.82 ± 0.46	N.M.	N.M.	0.04 ± 0.04	N.M.
Van Horne et al., 2021 [60]	Toenails; *n* = 95	Communities near metalworking industries in Los Angeles, USA	N.M.	0.23 ± 0.20	0.05 ± 0.08	N.M.	N.M.	N.M.	N.M.	1.72 ± 1.65	N.M.	N.M.	0.84 ± 0.89	0.94 ± 0.52	N.M.	N.M.	0.15 ± 0.13	N.M.
Yoo et al., 2002 [63]	Toenails; *n* = 150	Distribution of heavy metals in autopsy materials from normal Korean	176 ± 94	10 ± 13	0.7 ± 0.9	N.M.	2.9 ± 2.9	8.8 ± 7.0	141 ± 99	2.9 ± 2.8	1.9 ± 2.5	5.5 ± 5.8	12 ± 11	6.9 ± 7.9	N.M.	N.M.	2.7 ± 2.8	97 ± 38
Di Ciaula et al., 2020 [61]	Toenails; *n* = 158	People residing in an unpolluted area in Forlì, Italy	103.24 ± 11.01	0.00	0.07 ± 0.02	0.00	1.28 ± 0.44	4.74 ± 0.36	164.49 ± 21.06	2.47 ± 0.35	0.00	0.43 ± 0.18	0.95 ± 0.47	0.01 ± 0.003	0.00	0.00	0.02 ± 0.02	95.30 ± 3.09
Sureda et al., 2017 [59]	Toenails; *n* = 100	Unpolluted area in Mallorca, Spain	N.M	N.M	N.M	0.01	0.55	N.M	14.0	N.M	N.M	0.99	N.M	0.57	N.M	N.M	N.M	106.3

Data shown are AM ± SD, arithmetic mean ± standard deviation. N.M., not measured. ^a^ Number of participants in the study.

## Data Availability

The data presented in this study are available on request from the corresponding author. The data are not publicly available due to ethical considerations.
